# Exposure to Vitamin D Fortification Policy in Prenatal Life and the Risk of Childhood Asthma: Results from the D-Tect Study

**DOI:** 10.3390/nu11040924

**Published:** 2019-04-24

**Authors:** Fanney Thorsteinsdottir, Ekaterina Maslova, Ramune Jacobsen, Peder Frederiksen, Amélie Keller, Vibeke Backer, Berit Lilienthal Heitmann

**Affiliations:** 1 Fanney Thorsteinsdottir, Research Unit for Dietary Studies, The Parker Institute, Bisbebjerg og Frederiksberg Hospital, Nordre Fasanvej 57, 2000 Frederiksberg, Denmark; ramune.jacobsen@sund.ku.dk (R.J.); peder.frederiksen@regionh.dk (P.F.); amelie.cleo.keller@regionh.dk (A.K.); berit.lilienthal.heitmann@egionh.dk (B.L.H.); 2 Department of Primary Care and Public Health, Imperial College London, London W6 8RP, UK; ekaterina.maslova14@imperial.ac.uk; 3 Centre for Fetal Programming, Department of Epidemiology Research, Statens Serum Institut, 2300 Copenhagen, Denmark; 4 Department of Pharmacy, University of Copenhagen, 2100 Copenhagen, Denmark; 5 Department of Respiratory Medicine, Bispebjerg University Hospital, 2400 Copenhagen, Denmark; backer@dadlnet.dk; 6 The Boden Institute of Obesity, Nutrition, Exercise & Eating Disorders, University of Sydney, Sydney, NSW 2006, Australia; 7 The Department of Public Health, Section for General Practice, University of Copenhagen, 2100 Copenhagen, Denmark

**Keywords:** asthma, fortification, vitamin D, social experiment

## Abstract

Prenatal vitamin D insufficiency may be associated with an increased risk of developing childhood asthma. Results from epidemiological studies are conflicting and limited by short follow-up and small sample sizes. The objective of this study was to examine if children born to women exposed to the margarine fortification policy with a small dose of extra vitamin D during pregnancy had a reduced risk of developing asthma until age 9 years, compared to children born to unexposed women. The termination of a Danish mandatory vitamin D fortification policy constituted the basis for the study design. We compared the risk of inpatient asthma diagnoses in all Danish children born two years before (*n* = 106,347, exposed) and two years after (*n* = 115,900, unexposed) the termination of the policy. The children were followed in the register from 0–9 years of age. Data were analyzed using Cox proportional hazards regression. The Hazard Ratio for the first inpatient asthma admission among exposed versus unexposed children was 0.96 (95%CI: 0.90–1.04). When stratifying by sex and age, 0–3 years old boys exposed to vitamin D fortification showed a lower asthma risk compared to unexposed boys (HR 0.78, 95%CI: 0.67–0.92). Prenatal exposure to margarine fortification policy with extra vitamin D did not affect the overall risk of developing asthma among children aged 0–9 years but seemed to reduce the risk among 0–3 years old boys. Taking aside study design limitations, this could be explained by different sensitivity to vitamin D from different sex-related asthma phenotypes in children with early onset, and sex differences in lung development or immune responses.

## 1. Introduction

Asthma is one of the most common chronic conditions among children [1]. It is a complex heterogeneous disease that affects both the respiratory and the immune system [2]. It manifests by many phenotypes that vary by sex, age at onset, presence of obesity, as well as the severity of atopy, allergic sensitization, bronchial obstruction, and hyperresponsiveness. Although genetic factors and childhood exposure to environmental triggers, such as tobacco smoke, air pollution, viral infections or aeroallergens play a major role in the development of childhood asthma [3], it has been suggested that environmental exposures during gestation may also be important [4]. Asthma is more prevalent among boys until puberty when a shift towards higher prevalence among girls and women is observed [5,6]. In children, this is thought to be due to sex differences in lung development and inflammatory profile [7].

Vitamin D is a fat-soluble vitamin and a secosteroid hormone playing an important role in both skeletal and non-skeletal functions [8]. It readily crosses the placenta and the fetal supply is totally dependent on the supplies of the mother [9]. Results from animal and human studies have shown that prenatal vitamin D insufficiency may influence both the intrauterine immune system [10] and lung development [11,12], causing permanent changes to these systems. Such changes constitute a plausible biological basis to suggest that vitamin D insufficiency during gestation may have a programming effect contributing to the risk of childhood asthma. This is especially relevant given the high prevalence of vitamin D insufficiency in pregnant women [13]. The research on the association between prenatal vitamin D status and childhood asthma risk in the offspring is quite extensive. Results from several systematic reviews and meta-analyses of observational studies, however, have been inconsistent [14,15,16,17,18]. Two randomized clinical trials (RCT) have recently been conducted; both found non-significant 20% reduced risk of asthma/recurrent wheeze among 0–3 year old children whose mothers were supplemented with vitamin D during pregnancy [19,20], while a combined analysis of the two trials showed a significant reduction in offspring’s asthma/recurrent wheeze risk after vitamin D supplementation in pregnancy [21]. Despite sex differences in asthma prevalence and possible sex difference in the effect of vitamin D on asthma development, few observational studies and none of the RCTs have run analyses differentiated by sex. Furthermore, most of the previous studies had limited numbers of participants and short follow-up periods [14,15,16,17,18,21].

In Denmark, between 1937 and 1985, it was mandatory to fortify margarine with 1.25 µg vitamin D per 100 g [22]. The fortification accounted for on average 13% (3–29%) of total vitamin D intake from food in the Danish population [22]. Despite that, the mandatory margarine fortification policy was canceled in June 1985. This study utilized the design of this societal experiment grounded on the distinct in time termination of the Danish margarine vitamin D fortification policy. The objective of the present study was to examine if children born to women exposed to the margarine fortification policy with a small dose of extra vitamin D during pregnancy had a reduced risk of developing asthma until age 9 years compared to children whose mothers were unexposed to the fortification policy during pregnancy. Furthermore, this study examined whether the association between exposure and asthma risk varied by age, sex, and month of birth.

## 2. Methods

### 2.1. Study Design

**Figure 1:**
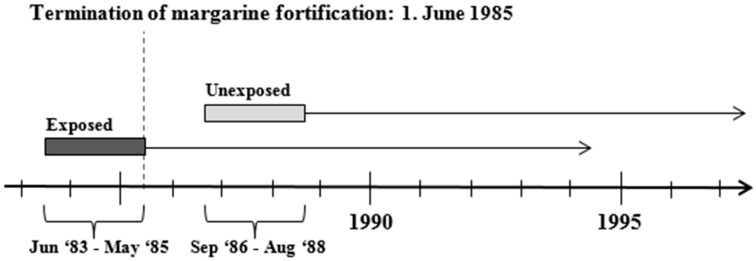
Study design and study population.

This study is a part of the D-tect project and a detailed study design description has been published elsewhere [23]. Briefly, all individuals born in Denmark during the two years before the termination of the vitamin D policy in 1985 were considered as exposed to vitamin D fortification during prenatal life, and all individuals born during the two years after the termination (excluding a wash-out period), were considered unexposed to vitamin D fortification. The washout period consisted of 9 months for the duration of a full-term pregnancy and an additional 6 months to allow for fortified products to be commercially replaced by non-fortified products (Figure 1).

All children born alive in Denmark from June 1983–May 1985 and from September 1986–August 1988 were identified using the Danish Civil Registration System (CRS) and included in this study. The CRS was established in 1968 and includes information on all individuals alive and with permanent residence in Denmark at that time, and those who were born in or immigrated to Denmark afterwards [24]. All individuals in Denmark are assigned a unique identification number (CPR number) that can be used to identify the individual in all national registers and databases. From the CRS we retrieved information on the date of birth, date of death (if any), date of emigration or lost to follow up (residence unknown to Danish authorities).

Individuals from our study population were followed in the Danish National Patient Register (DNPR) to identify childhood asthma diagnoses. The DNPR is a national administrative register established in 1977 that contains information on all hospital admissions, including discharge diagnoses, according to the international classification of diseases (ICD) system [25]. Outpatient admissions in DNPR were systematically registered from 1995, and to ensure comparable exposure groups with complete follow-up among both exposed and unexposed individuals we only analyzed inpatient asthma admissions in this study.

According to Danish law, ethical approval is not required for register-based studies. Permission to access data was granted by Forskerservice, Statens Serum Institut (J.no. FSEID-00001369). The Danish Data Protection Agency provided permission to process data (J.no. 2012-41-1156). The study is registered at ClinicalTrials.gov (NCT03330301).

### 2.2. Definition of Outcome

Childhood asthma was defined as asthma diagnoses from birth to the age of 9 years. We considered puberty as a cut-off for distinguishing childhood asthma from adult asthma, as puberty is the period when there is a sex-based shift in the prevalence of asthma from male predominance to female predominance. In a previous study conducted among Danish children aged 6.0–19.9 years, the mean age for the pre-pubertal stage was 10.88 (SD ± 8.66–13.11) years for girls and 11.83 (SD ± 9.92–13.75) for boys [26], defining the age of 9 as a certain cut-off for pre-pubertal stage.

Asthma was defined based on ICD-8 codes 493.00, 493.01, 493.08 and 493.09; and from 1994 onwards on ICD-10 codes DJ45, DJ45.0, DJ45.1, DJ45.8, DJ45.9, DJ46.0, and DJ46.9. The registry diagnoses of asthma have been previously validated against medical records [27].

### 2.3. Statistical Analysis

We conducted a time-to-event analysis focusing on the time of first inpatient asthma diagnosis. If asthma was not diagnosed at the age of 9, or if the child became inactive in the CRS (emigrated, lost to follow up or dead) before the age of 9, censoring took place. We used a Cox proportional hazard model with age as the underlying time scale to assess the hazard ratios of first inpatient asthma diagnosis in the group that was exposed to extra vitamin D from fortification during gestation compared to the group that was unexposed to extra vitamin D from fortification. The assumption of proportional hazard was examined using Schoenfeld residuals [28]. Data were presented as the time at risk, incident rate, and hazard ratio (95% confidence interval). Descriptive statistics were presented in frequencies (N) and percentages (%). In multivariable analysis, we adjusted for sex and month of birth. As we included entire birth cohorts of all individuals born in Denmark in adjacent years around the fortification termination, other potential confounders were considered to be equally distributed in both exposure groups, thus adjustment for other confounders was not deemed necessary.

To test our hypothesis that the greatest risk reduction would be observed among those who had most of their prenatal period during the darkest months, we examined if the effect of vitamin D fortification on asthma risk was modified by month of birth by the likelihood ratio test; the statistical tests were two-sided at a 5% significance level. In addition, as decided a priory, we conducted analyses stratified by sex and age at the time of asthma diagnosis hypothesizing potential effect modification by sex and age, since the prevalence of asthma is higher among boys especially in the first few years of life, and both sex and age are important characteristics of different asthma phenotypes [29].

All data management and descriptive statistics were performed using Stata 13 (StataCorp. 2013. Stata Statistical Software: Release 13. College Station, TX, USA, www.stata.com), whereas all statistical analyses were performed using R version 3.3.3 (R Foundation for Statistical Computing, Vienna, Austria, www.R-project.org).

## 3. Results

**Figure 2:**
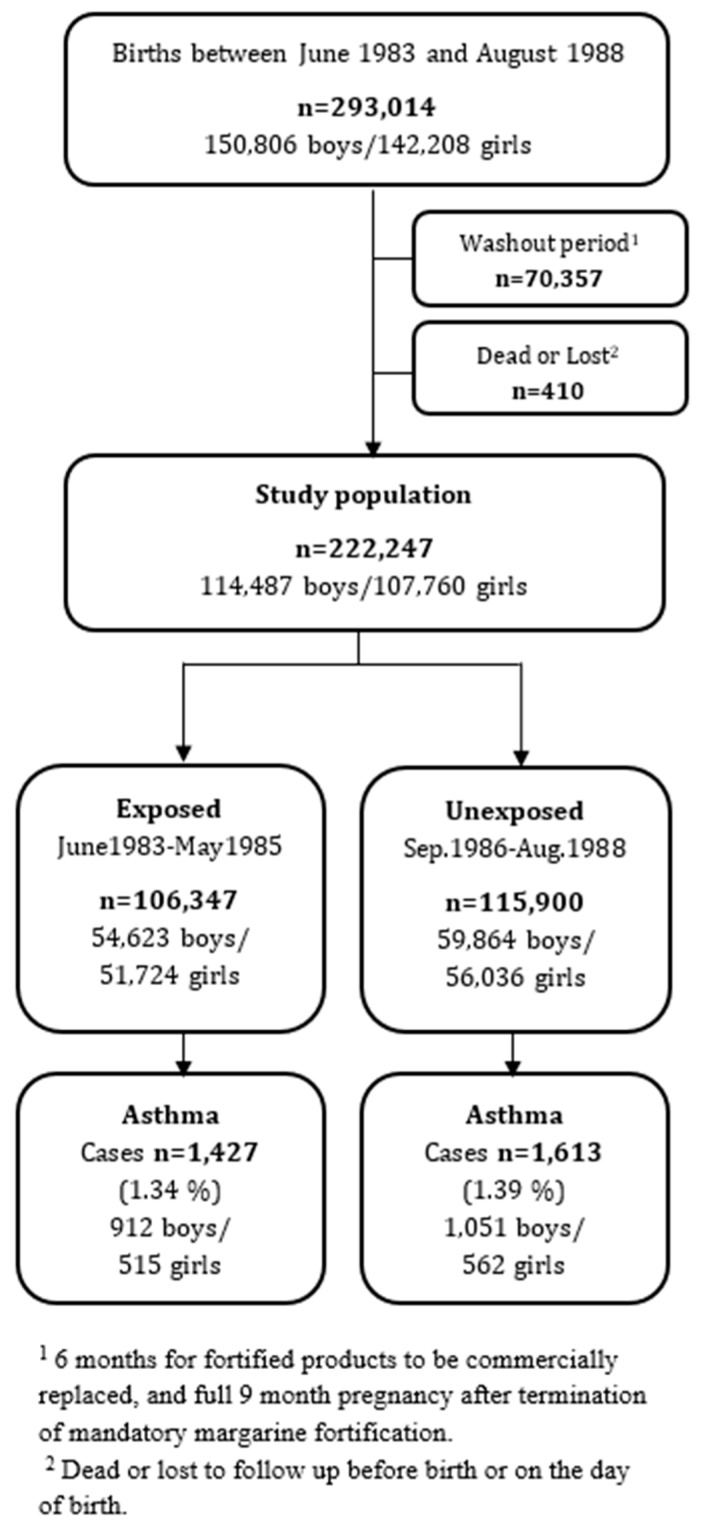
Flowchart of the study population.

Out of 222,247 children included in the study, 106,347 were born to mothers exposed to the margarine fortification policy with extra vitamin D during pregnancy; 115,900 were born to unexposed mothers. Among the exposed children, 1427 (64% boys) had inpatient asthma admission before the age of 9 years; and among unexposed children, 1613 (65% boys) had inpatient asthma admission before the age of 9 years (Figure 2 and Supplementary Table S1).

**Figure 3:**
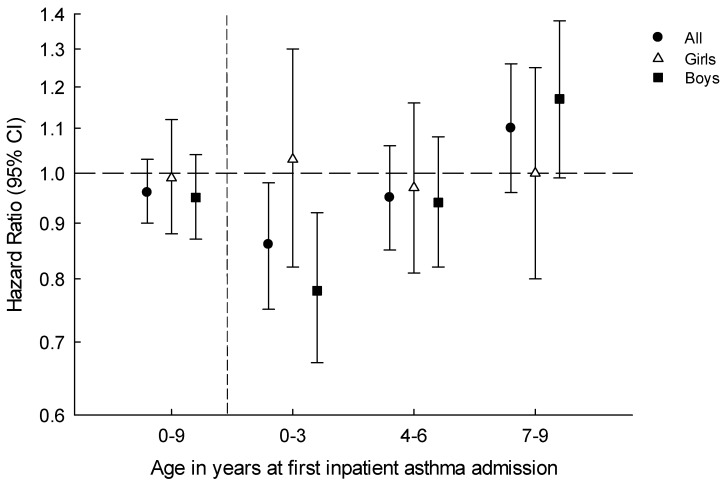
Hazard ratio of childhood asthma among those prenatally exposed to the margarine fortification with vitamin D policy, compared to those unexposed.

We did not observe a difference in inpatient asthma admission risk between children exposed and unexposed to the margarine fortification policy with extra vitamin D during the prenatal period (HR 0.96, 95% CI: 0.90–1.03). Furthermore, there was no effect modification by month of birth (*p* = 0.28). The Schoenfeld residuals indicated violation of the proportional hazards assumption with respect to exposure status, and when stratified by age at first diagnoses, we found that among the 0–3 years old, those exposed to extra vitamin D from fortification were less likely to have an inpatient asthma admission (HR 0.86, 95% CI: 0.75–0.98) compared to unexposed ones. Further stratification by sex revealed that reduced risk was confined to the 0–3-year-old boys. Boys of 0–3 years exposed to the margarine fortification policy with extra vitamin D were less likely to have an inpatient asthma admission (HR 0.78, 95% CI: 0.67–0.92) compared to unexposed ones. Adjusting for sex and month of birth gave similar results (Table 1 and Figure 3).

## 4. Discussion

Overall, we did not see a difference in the risk of asthma between children of both sexes age 0–9 years born to mothers exposed to the margarine fortification policy with a small extra dose of vitamin D during their entire pregnancy compared to those who were born to mothers who were unexposed. However, our analysis indicated that young children (0–3 years old), and in particular boys born to exposed mothers, had more than 20% lower hazards for developing asthma compared to boys born to unexposed mothers.

Several observational studies have examined the association between prenatal vitamin D and the development of asthma in the offspring, however, the results have been mixed. There are several potential reasons for this inconsistency. The observational studies have differed in study design, number of participants, adjustment for covariates, the time at exposure assessment, the source material for biomarker analysis, the concentration of maternal or cord blood 25(OH)D, and the assay method. Early observational studies looking at vitamin D intake during pregnancy, usually assessed by food frequency questionnaire (FFQ), and later development of asthma and wheezing, tended to find inverse associations [30,31,32]. Vitamin D may have served as a marker of a healthier diet in general and other dietary-related factors such as differences in the maternal microbiome [33]. The cohort studies examining maternal or cord blood 25(OH)D tended to find no association between 25(OH)D level in pregnancy and offspring asthma [34,35,36]. This was true across different timing of exposure and outcome assessment as well as geographical settings. Total 25(OH)D level may not be a sufficient indicator of the biologically available vitamin D, as other factors such as level of vitamin D binding protein could influence the metabolism and bioavailability [37,38]. These studies also did not look at the difference in asthma risk between different sex nor stratify according to pre-pregnancy vitamin D status which may be more important for reducing asthma risk than vitamin D status in pregnancy. In two recent randomized trials where mothers were supplemented with daily dose of 60 and 100 µg vitamin D respectively during pregnancy [19,20,21], inverse associations between 25(OH)D level and asthma/recurrent wheeze were strongest among women with a high 25(OH)D status at baseline (≥75 nmol/L). This could indicate that the programming effect already takes place in early pregnancy or even prior to pregnancy, and therefore, having optimal vitamin D status already at conception might be important for reducing the risk of childhood asthma. This may be supported by the results from observational studies assessing vitamin D intake as women with higher vitamin D intake during pregnancy are likely to have had the same dietary pattern prior to pregnancy and thus have higher vitamin D status at conception. In our study, the mothers were exposed to extra vitamin D from fortification both during a long period prior to pregnancy and during the entire pregnancy. Thus, it is expected that the exposed mothers had a higher vitamin D status pre-pregnancy than unexposed mothers. It is important to stress that the vitamin D dose administered to the subjects in the two trials was substantially higher than what women were exposed to from fortification in our study. We have previously calculated, based on the fortification dose (1.25 µg/100g) and margarine intake statistics, that an average additional 0.4–0.6 µg of vitamin D per day could be provided by the fortified margarine [39]. Compared to vitamin D supplementation in the two trials (i.e. 60 and 100 µg), and in light of the recommended total intake of 10 µg vitamin D per day for skeletal actions [40], the Danish margarine fortification policy provided a very low extra vitamin D dose. However, the optimal vitamin D dose for the prenatal development of the immune system and/or lung development is unknown. Much higher supplementation doses provided in the two previously mentioned trials seemed to be inadequate to reach a level of sufficiency among women during their pregnancy to prevent asthma development in the offspring. In our study, a constant intake of a very low extra dose of vitamin D via fortified foods consumption over entire pregnancy (and before pregnancy), might have been sufficient to reduce the risk in the offspring with the highest risk of developing asthma, the youngest boys [7,41]. Methodological advantages and disadvantages of our societal experiment design study, if compared to clinical trials, are discussed below under strengths and limitations.

Asthma phenotypes are defined based on age at debut, the presence of atopy, allergic sanitization, lung function, responsiveness to steroids, obesity, sex, and inflammatory profile. Many of these different phenotypes vary according to age, making age an important parameter in identifying asthma phenotypes [42]. Therefore, to try to isolate specific phenotypes, we stratified the analysis by age at onset. Our results of a slight reduction in the risk of inpatient asthma admission among 0–3 years old boys born to mothers who were exposed to the margarine fortification policy with extra vitamin D during pregnancy are in accordance with the results from the combined analysis of the two recently published RCTs showing a 26% reduction in the risk of asthma/recurrent wheeze at age 0–3 years among those born to mothers who took vitamin D supplementation during pregnancy [21]. This could indicate that vitamin D has an effect on asthma phenotypes that are prevalent in the youngest children, especially boys, or that the programming effect is relatively weaker than the effect of the risk factors accumulating during childhood. On the other hand, among children younger than 6 years, and particularly those younger than 3 years, the asthma diagnosis is based on symptoms (wheezing, cough, and breathlessness), clinical history of these symptoms, or family history of asthma or atopy, whereas among older children and adults asthma is diagnosed based on symptoms and confirmed with objective measures (spirometry, beta2 agonist reversibility test, bronchial provocation test, and peak flow measurements) [3]. Wheezing is the most common clinical manifestation of asthma onset. As very young children who experience severe and persistent wheezing are more likely to also have asthma in childhood or adulthood [43,44], persistent wheezing in very young children usually is considered to be an asthma indication. At the same time, many young children often experience wheezing due to viral respiratory tract infections (RTI) without having or later developing asthma [44]. Notably, a recent review on the association between maternal vitamin D status and RTI in offspring showed an inverse association between exposure to vitamin D and RTIs (highest vs. lowest 25(OH)D level: OR 0.64, 95% CI: 0.47–0.87), but no association with asthma or wheezing [18]. Hence, it cannot be ruled out that the protective effect observed among the 0–3-year-olds in our study was driven by a proportion of children misdiagnosed with asthma because of wheezing due to viral RTI.

In regard to the sex differences, there are anatomic differences in lung size, maturity, and function between the sexes with boys having larger lungs than girls, however, girls have higher forced expiratory flow rates [7]. The lungs of newborn boys are also less mature than the lungs of newborn girls [45] and thus are more vulnerable to respiratory infection, and consequently, asthma. Moreover, severe allergic asthma, or asthma with multiple sensitizations to allergens, characterized by early onset, high eosinophil count, and a low response to corticosteroid, is more prevalent among boys [29]. Children included in our study were children with asthma diagnosed during hospitalization, thus our outcome measure captured the more severe cases of asthma. It is therefore possible that the protective association observed among the youngest boys in our study was driven by the effects on an allergic type of asthma. Interestingly, despite sex differences in the prevalence of asthma, and different asthma phenotypes in boys and girls [44], very few studies have examined whether the effect of prenatal vitamin D exposure on the development of asthma is sex-specific. We identified only one other study that conducted sex-stratified analyses, and similarly to the results of the present study, the authors found an inverse association between maternal vitamin D and asthma risk at 6 years among boys only [46]. Consequently, vitamin D status in fetal life may contribute differently to the regulation of the immunologic responses in boys and girls.

Based on the present study, we cannot conclude that exposure to the margarine fortification policy with extra vitamin D during the prenatal period influences the risk of offspring asthma. However, our results and those of prior studies may indicate that vitamin D intake, even in small amounts, early in pregnancy or even before conception could influence the risk of asthma development, especially among more vulnerable groups such as very young male children. Future studies of the association between prenatal vitamin D and offspring asthma risk should assess vitamin D status or intervene with vitamin D supplements periconceptually or prior to pregnancy. Furthermore, they should focus on different phenotypes of asthma, using biomarkers shown to have a good prognostic value, and assessment of possible sex differences.

### Strengths and Limitations

In this study we utilized a societal experiment of an abrupt abolishment of an obligatory vitamin D fortification, that exposed all individuals living in Denmark to extra vitamin D from fortified margarine during a distinct period of time when the fortification policy was implemented, and not thereafter. A semi-ecological study design has both strengths and limitations.

The strength is that we could include all individuals born in Denmark from entire birth cohorts from the Danish population, capturing all inpatient asthma admissions, which makes our results generalizable for the entire Danish population. This was possible due to the excellent Danish administrative and health registers, extensively used in Danish epidemiological research [47,48]. Additionally, asthma diagnoses in DNPR have been previously validated [27]. Another strength of the design was that potential confounding (i.e., from differences in socioeconomic status, obesity, maternal lifestyle and diet during pregnancy) can be considered to be equally distributed in both the exposed and the unexposed groups as they included all individuals born in adjacent birth cohorts around the time of the policy change. Nevertheless, secular trends in potential confounders during this short period of time between 1983–1988 could have introduced residual confounding. Intake of margarine was remarkably stable during the years around the policy change, and we were not able to identify changes in national recommendations for intake and/or supplementation of vitamin D or in fortification practice. However, a study from Denmark shows that smoking among women, a risk factor for offspring asthma, decreased slightly during this period [49]. This would have attenuated our findings and therefore speaks in favor of a true association between exposure to the margarine fortification policy with extra vitamin D and the development of asthma. Furthermore, we know that overweight and obesity, a risk factor for asthma, has been increasing among women [50], and from 1987–1988 there were changes in fiscal policy in Denmark that caused economic challenges for many households [51,52]. It can be speculated that this economic crisis influenced overall diet quality, such as decreased the intake of fish or fish oil, which is also shown to have a protective effect on asthma development among offspring.

The limitation of this semi-ecological study design is that information on our exposure represents ecological data; we do not have any information on actual vitamin D intake, neither from the fortified margarine nor from other dietary sources and hence cannot make actual intake recommendations. A further limitation of the study is that we only included in-patient discharge diagnoses and not outpatients diagnoses and diagnoses by general practitioners, as the DNPR does not collect this information. Consequently, we have a subgroup of asthmatic children, most likely those with severe or uncontrolled asthma, and can generalize our results only to this group. Another study limitation related to the nature of register data, which does not specify different phenotypes of asthma, nor clinical parameters. To approximate this information, we conducted analyses stratified by age and sex; however, even though the phenotypes differ by age and sex, there are other parameters that are equally important, such as sensitization to allergens, responsiveness to steroids, and inflammatory profile that would also be interesting to examine.

## 5. Conclusions 

Our study, based on the societal experiment concerning margarine fortification with vitamin D in Denmark, suggests that prenatal exposure to a small dose of extra vitamin D from fortification may be associated with a lower risk of childhood asthma among boys aged 0–3 years, but not among older children or the youngest girls. Asthma phenotypes with very early onset that have different vitamin D sensitivity and/or sex differences in lung development or immune responses may explain our findings. However, residual confounding effects due to the semi-ecological design of the study cannot be ruled out.

## Figures and Tables

**Table 1:** Incidence, years at risk, rate, and hazard ratio (HR) of childhood asthma among those prenatally exposed to the margarine fortification with vitamin D policy, compared to those unexposed.

	Admissions	Time at Risk (Years)	Rate per 100,000 Years at Risk	Admissions	Time at Risk (Years)	Rate per 100,000 Years at Risk	HR	(95% CI)	Adjusted ^†^ HR	(95% CI)	*p* for Interaction with Month of Birth
	Exposed			Unexposed							
All	1427	938,797	152.0	1613	1,022,110	157.8	0.96	(0.90–1.03)	0.96	(0.90–1.04)	0.28
0–3 years	393	315,879	124.4	498	343,928	144.8	0.86	(0.75–0.98)	0.86	(0.75–0.98)	0.63
4–6 years	596	312,640	190.6	682	340,339	200.4	0.95	(0.85–1.06)	0.95	(0.85–1.06)	0.02
7–9 years	438	310,278	141.2	433	337,843	128.2	1.10	(0.96–1.26)	1.10	(0.96–1.26)	0.78
Girls	515	457,866	112.5	562	496,107	113.3	0.99	(0.88–1.12)	0.99	(0.88–1.12)	0.30
0–3 years	146	153,849	94.9	153	166,624	91.8	1.03	(0.82–1.30)	1.03	(0.82–1.30)	0.98
4–6 years	221	152,494	144.9	248	165,223	150.1	0.97	(0.81–1.16)	0.97	(0.81–1.16)	0.14
7–9 years	148	151,523	97.7	161	164,259	98.0	1.00	(0.80–1.25)	1.00	(0.80–1.24)	0.41
Boys	912	480,931	189.6	1,051	526,003	199.8	0.95	(0.87–1.04)	0.95	(0.87–1.04)	0.50
0–3 years	247	177,304	194.6	345	162,030	152.4	0.78	(0.67–0.92)	0.78	(0.67–0.92)	0.24
4–6 years	375	175,116	247.8	434	160,146	234.2	0.94	(0.82–1.08)	0.95	(0.82–1.09)	0.10
7–9 years	290	173,583	156.7	272	158,755	182.7	1.17	(0.99–1.38)	1.16	(0.99–1.37)	0.71

**^†^** = adjusted for sex and month of birth, except girls and boys are only adjusted for month of birth.

## Supplementary Materials

The following are available online at https://www.mdpi.com/2072-6643/11/4/924/s1, Table S1: Characteristics of the study population in numbers and percentages.
