# Supplementary material for: Exposure to Vitamin D Fortification Policy in Prenatal Life and the Risk of Childhood Asthma: Results from the D-Tect Study
Source: Nutrients. 2019 Apr 24;11(4):924. doi: 10.3390/nu11040924 (PMC6521227; doi:10.3390/nu11040924)
Supplement: Supplementary file 1 [file nutrients-11-00924-s001.pdf]

Supplementary table 1. Characteristics of the study population in numbers and percentages.

	Exposed (n=106,347)		Unexposed (n=115,900)	
	N	%	N	%
Girls	51,724	48.6	56,036	48.4
Boys	54,623	51.4	59,864	51.6
January	8,236	7.74	9,073	7.83
February	8,165	7.68	8,685	7.49
March	9,495	8.93	10,329	8.91
April	9,700	9.12	10,225	8.82
May	9,680	9.10	10,314	8.90
June	9,227	8.68	10,244	8.84
July	9,261	8.71	10,426	9.00
August	9,261	8.71	10,386	8.96
September	8,926	8.39	9,712	8.38
October	8,652	8.14	9,149	7.89
November	8,046	7.57	8,585	7.41
December	7,698	7.24	8,772	7.57
Asthma[†] cases	1,427	1.3	1,613	1.4
Girls	515	36.1	562	34.8
0–3 years	146	28.4	153	27.2
4–6 years	221	42.9	248	44.1
7–9 years	148	28.7	161	28.7
Boys	912	63.9	1,051	65.2
0–3 years	247	27.1	345	32.8
4–6 years	375	41.1	434	41.3
7–9 years	290	31.8	272	25.9
Censoring	104,920	98.7	114,287	98.6
Died	875	0.8	993	0.9
Lost to follow up	909	0.9	981	0.8
End of follow up	103,136	98.3	112,313	98.3

[†] Inpatient discharge diagnoses of asthma in the Danish National Patient Register.
